# Weighted gene co-expression network analysis identified six hub genes associated with rupture of intracranial aneurysms

**DOI:** 10.1371/journal.pone.0229308

**Published:** 2020-02-21

**Authors:** Qunhui Wang, Qi Luo, Zhongxi Yang, Yu-Hao Zhao, Jiaqi Li, Jian Wang, Jianmin Piao, Xuan Chen

**Affiliations:** Department of Neurosurgery, The First Hospital of Jilin University, Changchun, Jilin, P. R. China; Stellenbosch University Faculty of Medicine and Health Sciences, SOUTH AFRICA

## Abstract

Intracranial aneurysms (IAs) are characterized by localized dilation or ballooning of a cerebral artery. When IAs rupture, blood leaks into the space around the brain to create a subarachnoid hemorrhage. The latter is associated with a higher risk of disability and mortality. The aims of this study were to gain greater insight into the pathogenesis of ruptured IAs, and to clarify whether identified hub genes represent potential biological markers for assessing the likelihood of IA progression and rupture. Briefly, the GSE36791 and GSE73378 datasets from the National Center of Biotechnology Information Gene Expression Omnibus database were reanalyzed and subjected to a weighted gene co-expression network analysis to test the association between gene sets and clinical features. The clinical significance of these genes as potential biomarkers was also examined, with their expression validated by quantitative real-time PCR. A total of 14 co-expression modules and 238 hub genes were identified. In particular, three modules (labeled turquoise, blue, and brown) were found to highly correlate with IA rupture events. Additionally, six potential biomarkers were identified (*BASP1*, *CEBPB*, *ECHDC2*, *GZMK*, *KLHL3*, and *SLC2A3*), which are strongly associated with the progression and rupture of IAs. Taken together, these findings provide novel insights into potential molecular mechanisms responsible for IAs and they highlight the potential for these particular genes to serve as biomarkers for monitoring IA rupture.

## 1. Introduction

Intracranial aneurysms (IAs) represent a cerebrovascular disorder which affects between 3% and 5% of individuals. There is a potential risk that an IA will rupture, and this risk is higher in the posterior circulation [1–3]. With advances in intracranial imaging technologies, IAs have been detected more frequently. Consequently, clinicians are increasingly confronted with a dilemma regarding the choice of clinical management. Namely, whether preventive treatments (e.g., endovascular or surgical aneurysm repair) which are associated with inherent complication risks, or conservative management with or without follow-up imaging which leaves patients with a small, yet definite, risk of aneurysm rupture, should be applied [3, 4]. When an IA ruptures, a subarachnoid hemorrhage (SAH) develops. This life-threatening clinical condition has an acute mortality rate of approximately 50% [5, 6]. Despite considerable advances in therapy for IAs, SAH remains a highly lethal condition which is associated with a high socioeconomic burden [7–9]. Thus, an ability to identify IAs which have a high risk of rupture and provide timely preventive treatment may be key to the successful management of IAs.

To predict IA rupture, researchers have studied aneurysmal hemodynamics [10, 11], aneurysmal morphology and location [8], genetics [12], and other factors (e.g., cigarette smoking, hypertension, and positive family history for SAH) [13]. Only inflow angle was identified as a significant predictor of rupture according to morphological parameters [8]. However, aneurysm wall inflammation has also been shown to play a pivotal role in aneurysm growth and rupture [2, 3, 14]. Two scoring systems have been established to evaluate the risk of rupture and to guide treatment. These include the PHASES (population, hypertension, age, size of aneurysm, earlier SAH from another aneurysm, and site of aneurysm) system and the UIATS (unruptured IA treatment score) system [8, 15, 16]. However, standardized management of unruptured IAs remains controversial, as risks of prophylactic treatment must be weighed against possible risk of rupture for individual aneurysms [17].

**Fig 1:**
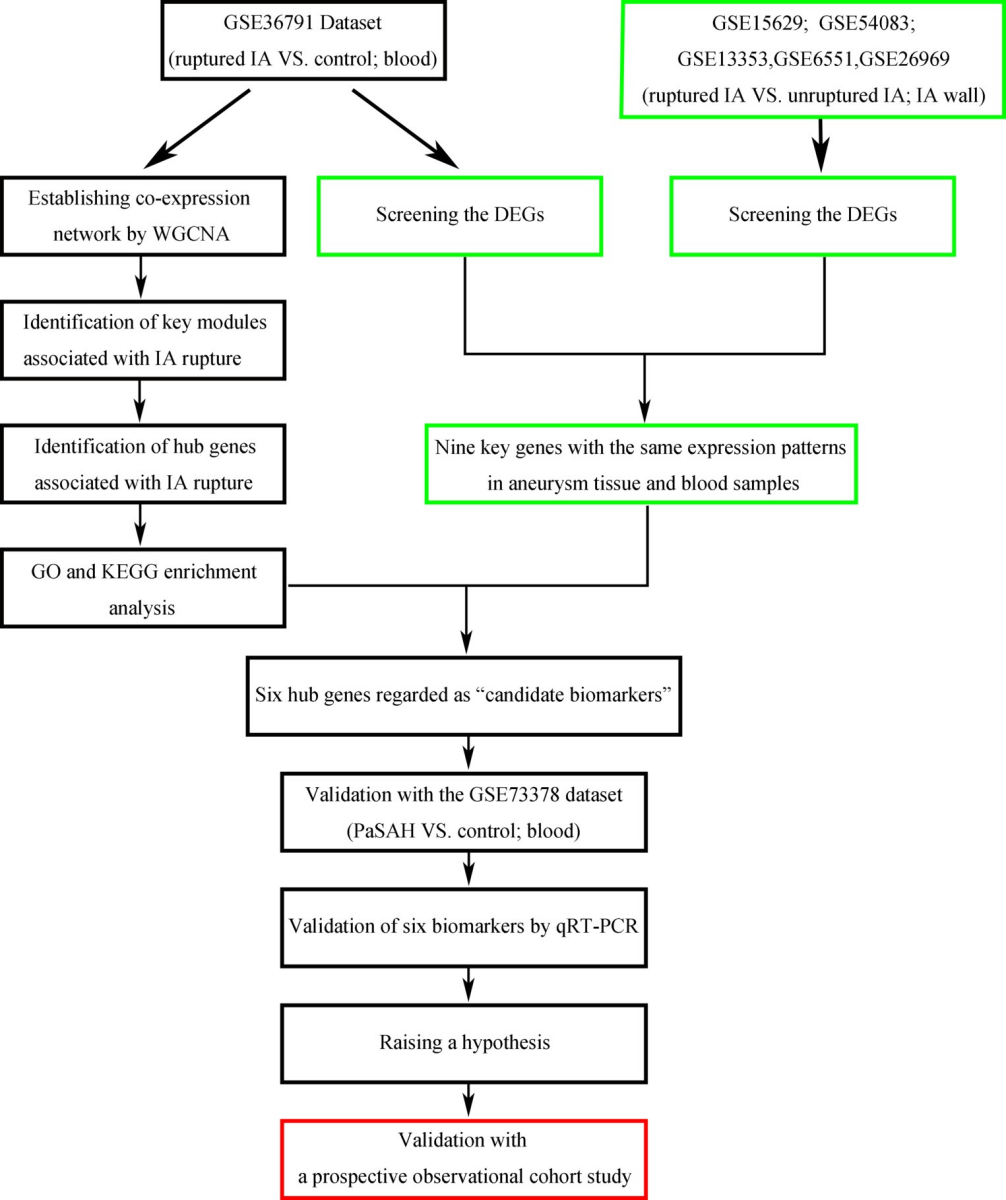
A flow chart illustrating the method used to identify six biomarkers associated with IA rupture. The entries shown in the green box were completed in our previous study, while the entries shown in the red box are those that we intend to focus on in future studies.

Despite the vast efforts made to date to prevent the rupture of IAs, the mechanisms mediating the pathology of IAs remains largely unknown. In particular, suitable biomarkers to predict IA rupture remain unavailable. Therefore, the aims of the present study were to gain greater insight into the pathogenesis of ruptured IAs and to clarify whether identified hub genes can be used as potential biological markers to assess the likelihood of IA progression and rupture (Fig 1). For these aims, the GSE36791 [18] and GSE73378 [19] datasets from the National Center of Biotechnology Information (NCBI) Gene Expression Omnibus (GEO, http://www.ncbi.nlm.nih.gov/geo/) were reanalyzed. A weighted gene co-expression network analysis (WGCNA) [20] was performed to test possible correlations between gene sets and clinical features of IAs. Clinically significant genes were identified and their expression levels were validated in patients with ruptured versus unruptured IAs by quantitative real-time (qRT)-PCR.

## 2. Materials and methods

### 2.1 Microarray dataset

Gene expression profile data from the GSE36791 dataset were used as a training set to construct co-expression networks and to identify hub genes. This dataset was generated from peripheral blood samples collected from 43 patients with SAH due to ruptured IAs, and from 18 individuals with headaches (the reference group) [18]. To verify the results obtained, the GSE73378 dataset was used as a test set. The GSE73378 dataset was generated from peripheral blood samples collected from 103 patients who developed an aneurysmal SAH at least two years prior, and from 107 individuals used as a reference group [19].

### 2.2 Data processing and WGCNA

The GSE36791 expression data were already normalized with a BeadArray package [21] (quantile normalization). Probe sets were then mapped to gene symbols according to the GPL10558 platform. After filtering the probes without a corresponding gene symbol, the average value of the gene symbols with multiple probes was calculated [22]. Outlier samples were identified with hierarchical cluster analysis (i.e., distance matrices constructed with Pearson's correlation matrices and a hierarchical agglomerative method to adopt average linkages) by using the hclust function in WGCNA [20]. Based on these results, three samples (GSM901111, GSM901112, and GSM901161) in GSE36791 were removed from subsequent analyses.

In the present study, the co-expression analysis performed was based on a WGCNA, which is a systems biology method for describing correlation patterns among genes across microarray samples [20]. Currently, this method is widely used in the international biomedical field. This method also helps identify clusters (modules) of highly correlated genes across samples. To identify ruptured IA-associated co-expression modules and their key constituents, preprocessed data of GES36791 were analyzed. Briefly, Pearson's correlation matrices were generated (average linkage method) for all pair-wise genes. Next, co-expression similarities were transformed into a weighted adjacency matrix of connection strengths by using a power adjacency function (cutoff height of 0.85). This adjacency matrix was then transformed into a topological overlap matrix to measure relative gene interconnectedness and proximity. Finally, gene co-expression modules were identified based on clustering (hierarchical average linkage) according to topology overlap.

### 2.3 Identification of modules with clinical significance

In order to identify modules related to the clinical traits of IA (group [rupture IA/ reference] and gender), two different approaches were used [20, 23]. The first approach was to determine gene significance (GS) from a linear regression analysis of gene expression data and clinical traits [20]. GS was defined based on a log10 transformation of P-values. Thus, a higher absolute value of GS_*i*_ corresponds with greater biological significance of gene *i* [20]. In addition, module significance (MS) is defined as the average GS value for all of the genes in a module, which implies that the higher the absolute value of MS_*j*_, the more biologically significant is module *j*. The second approach was to define module eigengenes (MEs) as a major component in the principal component analysis for each module. Thus, MEs can be considered representative of the gene expression profiles in a module [20]. Correlations between MEs and clinical traits were calculated to identify relevant modules. Modules with a P-value greater than 0.005 were considered to significantly correlate with certain clinical traits (ruptured IA).

### 2.4 Identification of hub genes and functional enrichment analysis

Hub genes were defined according to module connectivity [absolute value of Pearson's correlation (cor.geneModuleMembership > 0.8)] and clinical trait relationship [absolute value of Pearson's correlation (cor.geneTraitSignificance > 0.5)] [23]. Hub genes were highly interconnected with genes in the module with potential significance. To functionally characterize the identified hub genes, Gene Ontology (GO) terms and Kyoto Encyclopedia of Genes and Genomes (KEGG) pathway enrichment analysis were performed by using clusterProfiler [24]. The terms obtained from the KEGG pathway and GO analyses (including molecular functions, biological processes, and cellular components) which had false discovery rates (FDRs) < 0.05 were considered to be significantly enriched in the hub genes. Common hub genes were also screened from key genes identified in our previous study [22] which were regarded as “candidate biomarkers” highly associated with IA rupture.

### 2.5 Analysis of the GSE73378 dataset

GSE73378 expression data were already normalized (quantile normalization) by the surrogate variable analysis (SVA) package [19]. Differentially expressed genes (DEGs) (ruptured IAs vs. references) were then screened by using the limma package [25]. The Benjamini and Hochberg FDR [26] was used to adjust for multiple testing. Threshold values of FDR < 0.005 and absolute fold-change > 1.5 (|log_2_FC| > 0.585) were applied. Hierarchical clustering analyses (i.e., distance matrices constructed with Pearson's correlation matrices and a hierarchical agglomerative method to adopt average linkages) were also performed to validate the expression of hub genes regarded to be candidate biomarkers for IA rupture. Clustering heat maps were generated by using the pheatmap package [27] in R software (version 3.4.2).

### 2.6 Collection of blood samples

The expression profiles of six common genes considered to be possible biomarkers were validated in peripheral blood samples collected from 30 patients recruited from the Department of Neurosurgery, The First Hospital of Jilin University, China by qRT-PCR. Patient characteristics are provided in Table 1. Written consent was obtained from all participating individuals prior to sample collection and collection was approved by the Medical Ethics Committee of The First Hospital of Jilin University, in accordance with the Declaration of Helsinki.

**Table 1:** Clinical characteristics of the patient groups who provided blood samples for qRT-PCR analysis.

	Reference	Ruptured IA	PaSAH
Number	10	10	10
Age (mean ± SD), years	54.00 ± 6.88	51.70 ± 12.95	54.80 ± 10.76
Females (%)	50	60	60
History of smoking (%)	30	40	10
History of hypertension (%)	30	30	30
Excessive drinking (%)	20	20	0

Reference group: patients with unruptured IAs; Ruptured IA group: patients with ruptured IA; PaSAH group: patients with aneurysmal SAH within at least the prior year.

### 2.7 Validation of hub genes by qRT-PCR

Briefly, whole blood samples were homogenized in Trizol reagent (Invitrogen, Carlsbad, CA, USA) before total cellular RNA was extracted and transcribed to cDNA with a PrimeScript RT Reagent kit (Invitrogen). Quantitative PCR was subsequently performed by using Hieff^TM^ qPCR SYBR Green Master Mix (Low Rox Plus; Yisheng Biotech, Shanghai, China) with the MxPro-Mx3005 P Real-time PCR system (Agilent Technologies, Palo Alto, CA, USA). All primer oligos were synthesized by Sangon Biotechnology (Shanghai, China; listed in Table 2). Levels of mRNA expression were standardized to the mRNA level of glyceraldeyhyde 3-phosphate deydrogenase (*GAPDH*). Relative quantification was subsequently performed according to the comparative threshold cycle (2^-ΔΔCT^) method.

**Table 2:** Sequences of the qRT-PCR primers used in this study.

Gene	Direction	Sequence (5′-3′)
*BASP1*	Forward	GGAAGCGCCTAGTTCCACAC
	Reverse	TTGGTCGGAATTAGCTGCCG
*CEBPB*	Forward	AAGACCGTGGACAAGCACAG
	Reverse	AACAAGTTCCGCAGGGTGC
*ECHDC2*	Forward	CTTCGTCAGTGAGCTGCTGG
	Reverse	CACGCCCTTCACTCCACTTC
*GZMK*	Forward	ACCCTGCGAGAAGTCACTGT
	Reverse	CTGGCCTTTGGCATCTCCTG
*KLHL3*	Forward	GAGCAGTACAACCCAGCGAC
	Reverse	GTCCGCTAAGCACTCCAACC
*SLC2A3*	Forward	TTCAATGCTGATTGTCAACCTG
	Reverse	GCATTTCAACCGACTTAGCTAC
*GAPDH*	Forward	CCAAGGTCATCCATGACAACT
	Reverse	CAGGGATGATGTTCTGGAGAG

### 2.8 Statistical analysis

Experimental qRT-PCR data are presented as the mean ± standard error of the mean (SEM). R software (version 3.4.2) was used to perform statistical analyses. Differences between experimental groups (i.e., ruptured IA vs. reference group; past aneurysmal SAH (PaSHA) vs. reference group) were determined by applying unpaired Student’s *t*-tests.

## 3. Results

### 3.1 Weighted co-expression network construction

**Fig 2:**
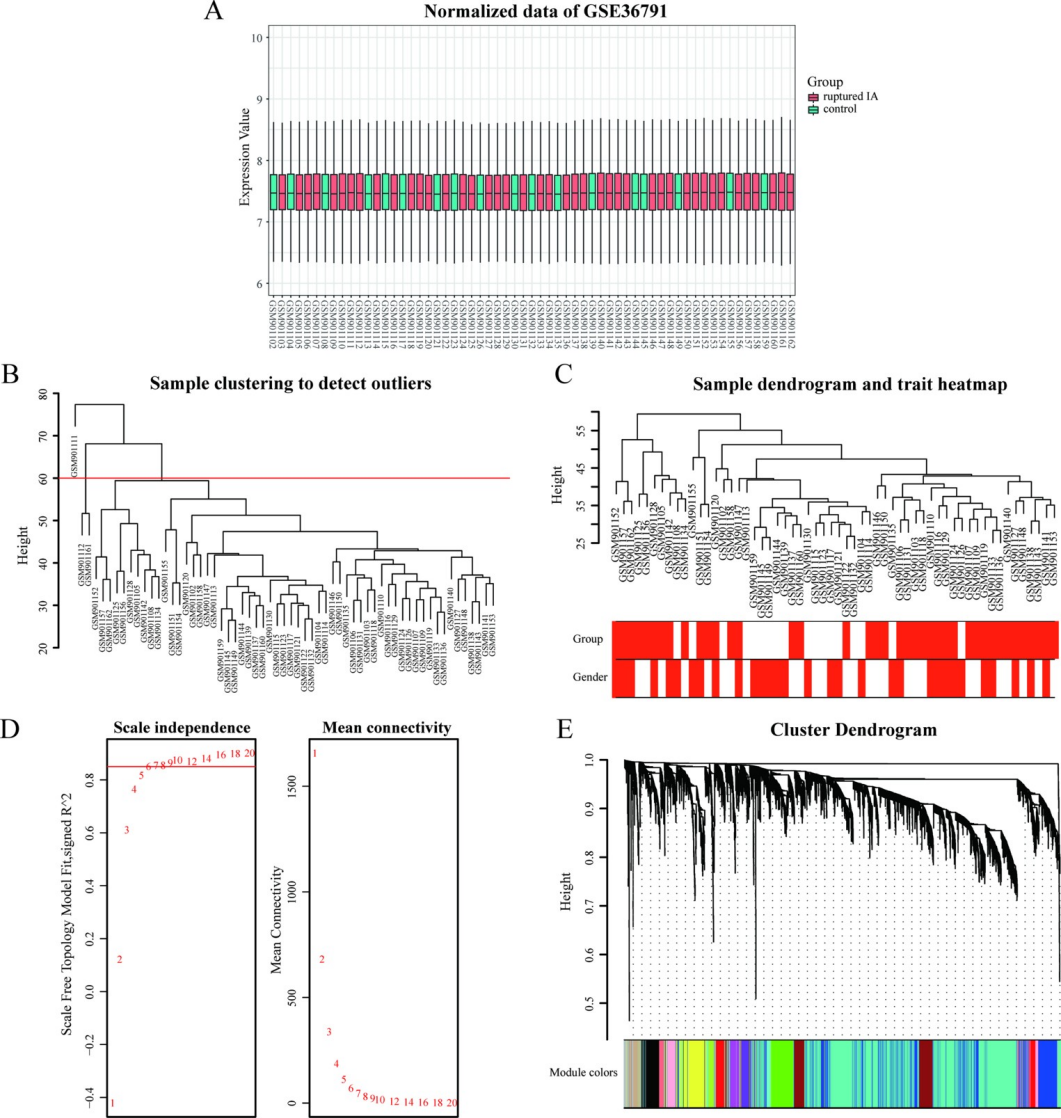
Expression analysis of the GSE36791 dataset. (A) Reliability of the data is shown with a box plot of the normalized data of GSE36791. (B) Sample clustering was performed to detect outliers. Three samples were excluded in this study (GSM901111, GSM901112, and GSM901161). (C) A clustering dendrogram of 58 samples and their associated clinical traits was generated based on gene expression. Color intensity is proportional to group (i.e., ruptured IA/reference groups) and gender. (D) The left and right panels show analyses of the scale-free fit index and mean connectivity for various soft-thresholding powers (β), respectively. (E) Modules associated with clinical information were identified and labeled with different colors.

The distribution of intensity values for the normalized expression data from all of the samples is shown in the box plot of Fig 2A. These results confirm the reliability of the data used. After preprocessing of the data, a total of 31,335 genes were obtained and 58/61 samples with clinical data were included in the co-expression analysis (Fig 2B and 2C). In the WGCNA [20], a soft threshold parameter, β, of the power function is used to ensure that the co-expression network (adjacency matrix) best approximates a scale-free topology [28]. In the present study, a β value of 6 (scale free R^2^ = 0.85) was used to ensure a scale-free network (Fig 2D). To identify a final set of hub genes, we first screened the top 25% of the 31,335 genes obtained according to variance to establish a co-expression network by using the “WGCNA” package [20] in R software. The genes which exhibited similar expression patterns were grouped into modules by applying average linkage hierarchical clustering. A total of 14 modules were identified and constructed. These modules are represented with different colors (Fig 2E).

### 3.2 Identification of key modules

**Fig 3:**
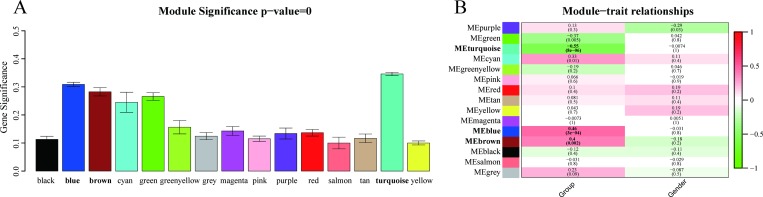
Identification of key modules. (A) The distribution of average gene significance and errors are shown for the modules associated with IA rupture. The turquoise, blue, and brown modules were identified as key modules. (B) A heatmap was generated to show correlations between module eigengenes and various clinical characteristics (e.g., gender and group). Corresponding correlation coefficients and P-values are also provided. Associated P-values for the turquoise, blue, and brown modules are indicated in parentheses and significant values are presented in bold font.

First, MS was used to test correlations between each module and IA rupture. As a result, five modules (out of 14 total modules) were found to exhibit markedly higher correlations with IA rupture [Fig 3A; turquoise module (R^2^ = 0.35), blue module (R^2^ = 0.31), brown module (R^2^ = 0.28), green module (R^2^ = 0.27), and cyan module (R^2^ = 0.25)]. The second method employing MEs showed that the turquoise, blue, and brown modules exhibited a significant correlation with IA rupture (P < 0.005, Fig 3B). Therefore, the turquoise, blue, and brown modules were identified as key modules and were subjected to further analysis.

### 3.3 Identification of hub genes and their functional annotations

**Fig 4:**
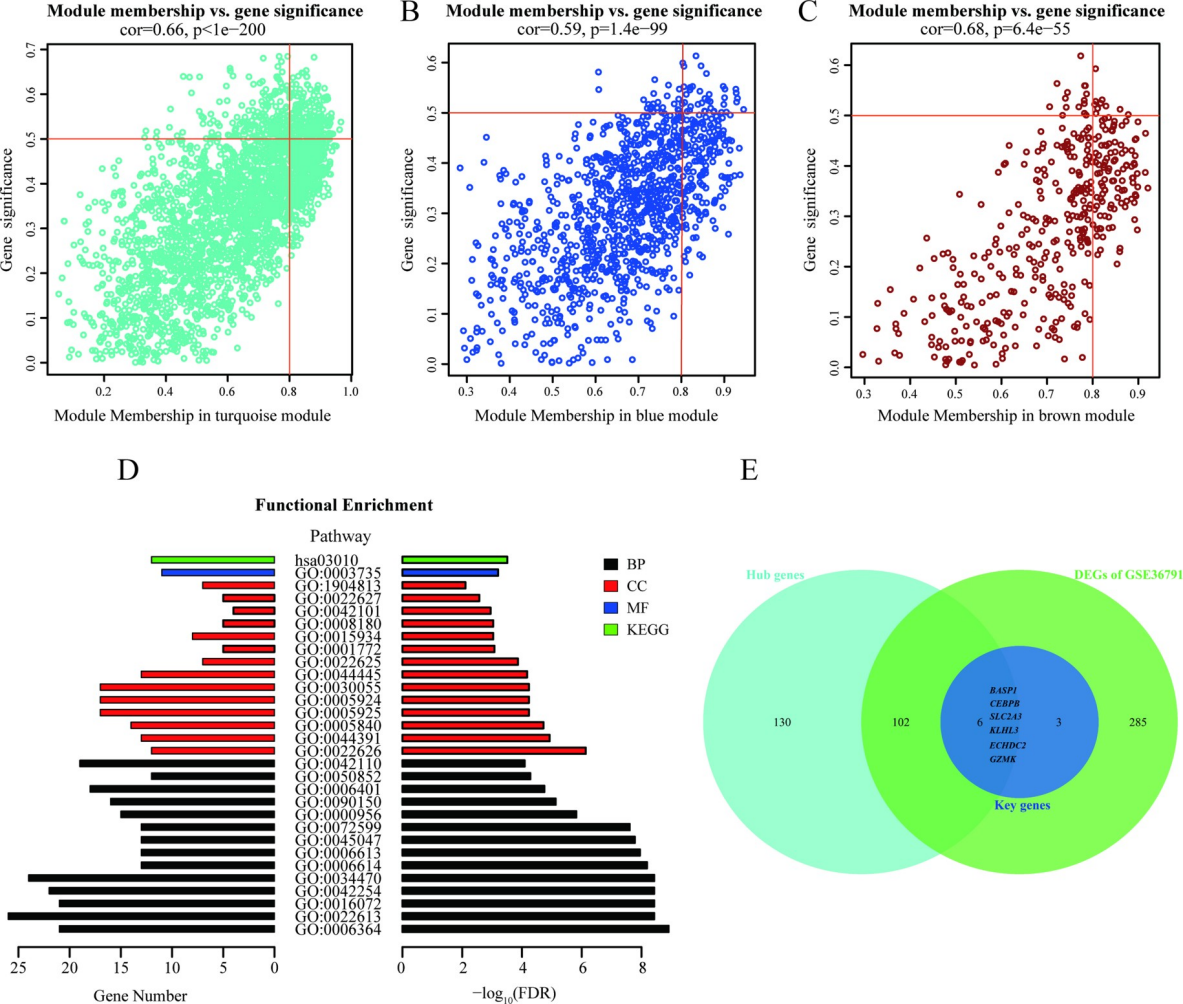
Detection and functional analysis of hub genes. Scatterplots were generated to depict correlations between module membership and gene significance values for the turquoise (A), blue (B), and brown (C) modules. Highly significant correlations were observed between gene significance and module membership in each of these three modules. (D) GO and KEGG annotations of the hub genes were performed. GO terms and KEGG pathways with threshold values of FDR < 0.05 were considered significant and are labeled as follows: BP (biological process) in black; CC (cellular component) in red; MF (molecular function) in blue; and KEGG (Kyoto Encyclopedia of Genes and Genomes) in green. (E) A Venn diagram displays the six common genes among the hub genes, key genes, and DEGs. Thus, these six genes are considered candidate biomarkers.

Hub genes are highly connected in a module and may be essential for certain biological processes. According to the module connectivity and clinical trait relationships evaluated in the present study (see Methods), a total of 238 genes exhibited high connectivity among three modules (190 genes in the turquoise module; 38 genes in the blue module; and 10 genes in the brown module). Consequently, these 238 genes were identified as hub genes (Fig 4A–4C). When all of these hub genes were subsequently subjected to a GO analysis, the most enriched GO terms were found to involve ribosomes and related pathways. Regarding the biological process (BP) terms for the hub genes, these were mainly enriched in rRNA processing, ribonucleoprotein complex biogenesis, rRNA metabolic process, and ribosome biogenesis; for the cellular compartment (CC) terms, the hub genes were mainly enriched in cytosolic ribosome, ribosomal subunit, ribosome, and focal adhesion (S1 and S2 Figs). Similarly, the KEGG pathway analysis also found the ribosome pathway to be enriched for the hub genes (Fig 4D). The top 30 enrichment GO/KEGG terms (listed according to FDRs and category) are presented in Table 3. In our previous study [22], we identified 396 DEGs in the GSE36791 dataset. Nine of these DEGs are considered to be key genes associated with IA rupture: *BASP1*, *CD74*, *CEBPB*, *ECHDC2*, *GZMK*, *HLA-DRB3*, *KLHL3*, *SLC2A3*, and *SOCS3* [22]. In the present study, the two approaches used to analyze the GSE36791 dataset identified 108 common genes among the hub genes, and also among the DEGs. In particular, six genes (*BASP1*, *CEBPB*, *ECHDC2*, *GZMK*, *KLHL3*, and *SLC2A3*) were included in both our previously published key genes (n = 9) [22] and in the 108 common genes identified from the GSE36971 dataset (Fig 4E). Therefore, these six genes were identified as potential candidate biomarkers and were subjected to further validation.

**Table 3:** The top 30 functional enrichment terms enriched for the hub genes identified in this study.

Category	ID	Term	Count	P-value	FDR
BP	GO:0006364	rRNA processing	21	1.23E-09	1.05E-09
BP	GO:0022613	Ribonucleoprotein complex biogenesis	26	3.75E-09	3.21E-09
BP	GO:0016072	rRNA metabolic process	21	3.75E-09	3.21E-09
BP	GO:0042254	Ribosome biogenesis	22	3.75E-09	3.21E-09
BP	GO:0034470	ncRNA processing	24	3.75E-09	3.21E-09
BP	GO:0006614	SRP-dependent cotranslational protein targeting to membrane	13	6.54E-09	5.59E-09
BP	GO:0006613	Cotranslational protein targeting to membrane	13	1.13E-08	9.64E-09
BP	GO:0045047	Protein targeting to ER	13	1.67E-08	1.43E-08
BP	GO:0072599	Establishment of protein localization to endoplasmic reticulum	13	2.47E-08	2.11E-08
BP	GO:0000956	Nuclear-transcribed mRNA catabolic process	15	1.51E-06	1.29E-06
BP	GO:0090150	Establishment of protein localization to membrane	16	7.43E-06	6.35E-06
BP	GO:0006401	RNA catabolic process	18	1.77E-05	1.51E-05
BP	GO:0050852	T cell receptor signaling pathway	12	5.27E-05	4.51E-05
BP	GO:0042110	T cell activation	19	8.12E-05	6.95E-05
CC	GO:0022626	Cytosolic ribosome	12	7.34E-07	5.92E-07
CC	GO:0044391	Ribosomal subunit	13	1.18E-05	9.49E-06
CC	GO:0005840	Ribosome	14	1.90E-05	1.53E-05
CC	GO:0005925	Focal adhesion	17	5.83E-05	4.69E-05
CC	GO:0005924	Cell-substrate adherens junction	17	5.83E-05	4.69E-05
CC	GO:0030055	Cell-substrate junction	17	5.83E-05	4.69E-05
CC	GO:0044445	Cytosolic part	13	6.70E-05	5.40E-05
CC	GO:0022625	Cytosolic large ribosomal subunit	7	1.36E-04	1.10E-04
CC	GO:0001772	Immunological synapse	5	8.29E-04	6.68E-04
CC	GO:0015934	Large ribosomal subunit	8	9.12E-04	7.35E-04
CC	GO:0008180	COP9 signalosome	5	9.12E-04	7.35E-04
CC	GO:0042101	T cell receptor complex	4	1.12E-03	9.06E-04
CC	GO:0022627	Cytosolic small ribosomal subunit	5	2.67E-03	2.15E-03
CC	GO:1904813	Ficolin-1-rich granule lumen	7	7.77E-03	6.26E-03
MF	GO:0003735	Structural constituent of ribosome	11	6.27E-04	5.28E-04
KEGG	hsa03010	Ribosome	12	3.09E-04	3.08E-04

BP: biological process; CC: cellular component; MF: molecular function; KEGG: Kyoto Encyclopedia of Genes and Genomes; FDR: false discovery rate.

### 3.4 Validation with the GSE73378 dataset

**Fig 5:**
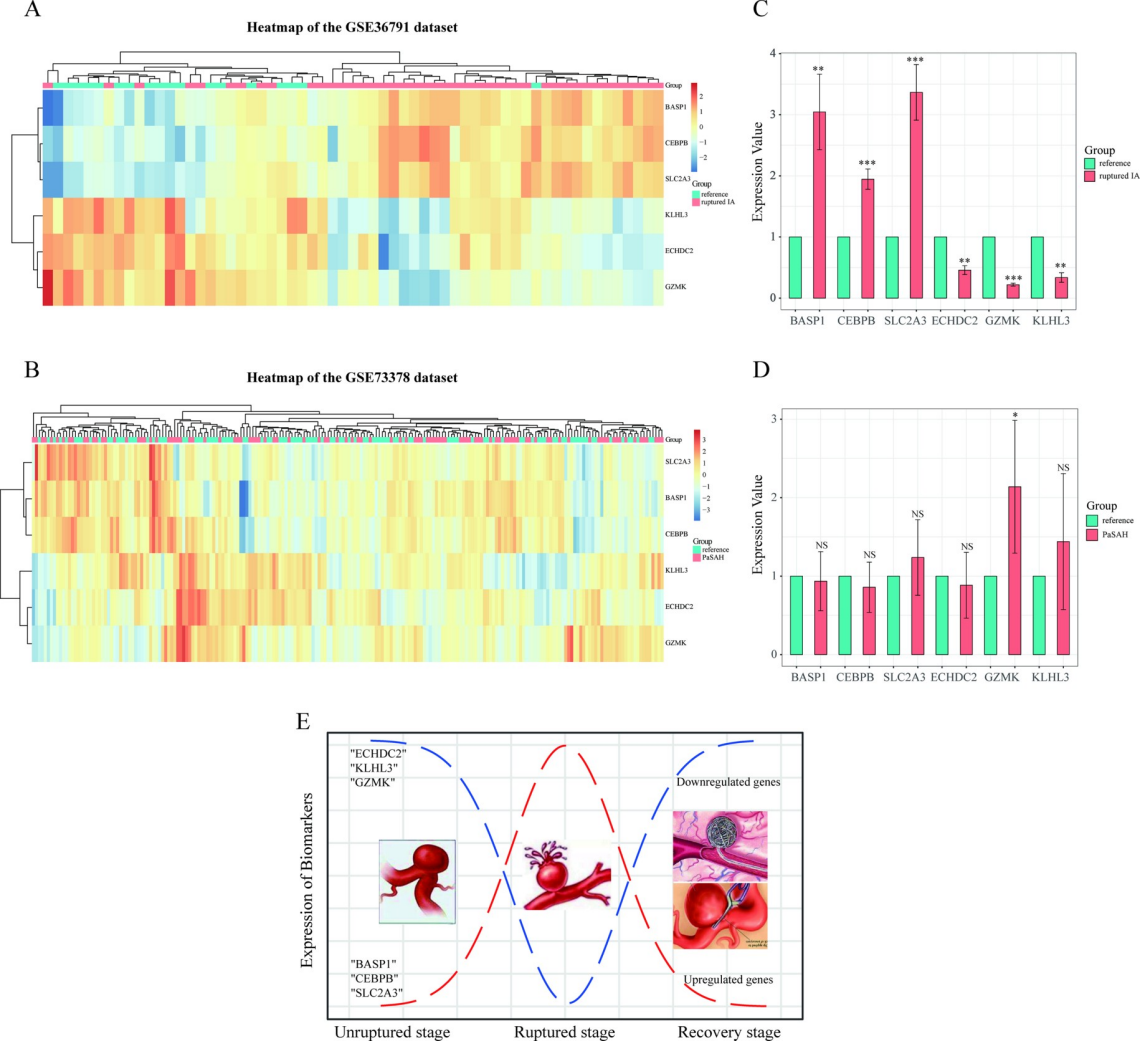
Heat maps show the expression profiles of the six candidate biomarker genes in the GSE36791 (A) and GSE73378 (B) datasets. Orange and blue coloring indicates high and low expression values, respectively. In the GSE36791 dataset, there is a mixture of ruptured aneurysm samples in the reference group. However, a good cluster was still obtained for the ruptured IA group compared to the GSE73378 dataset. (C, D) Validation of gene expression levels for *BASP1*, *CEBPB*, *SLC2A3*, *ECHDC2*, *GZMK*, and *KLHL3* in ruptured IA (or PaSAH) and reference samples. Data are presented as 2^-ΔΔCT^ relative to *GAPDH*. *P < 0.05, **P < 0.01, ***P < 0.001, NS: not significant. (E) A representation of the biological events which occur during IA rupture through recovery, with gene regulation of the six relevant genes identified in the present study shown as well.

The six candidate biomarkers were examined with normalized data of the GSE73378 dataset from the GEO database (Table 4). These data derived from 103 blood samples collected from patients with aneurysmal SAH at least two years prior, as well as from 107 blood samples collected from individuals included as a reference group. After data preprocessing, none of the genes exhibited a significant difference in expression (i.e., a FDR < 0.005 and an absolute fold-change > 1.5), consistent with the results of previous studies [19]. Consequently, hierarchical clustering analysis was performed for the six candidate biomarkers among samples of the GSE73378 dataset. No significant systematic variations in gene expression were found in the GSE73378 dataset, in contrast with the GSE36791 dataset (Fig 5A and 5B).

**Table 4:** Comparison of six hub genes between two datasets.

Hub Gene	GSE36791 Dataset			GSE73378 Dataset	
	Module	log_2_ FC	FDR	log_2_ FC	FDR
*BASP1*	Blue	0.741	3.24E-06	0.088	0.20
*CEBPB*	Blue	0.608	3.88E-05	0.078	0.18
*ECHDC2*	Turquoise	-0.796	1.12E-05	-0.058	0.20
*GZMK*	Blue	-0.980	2.23E-06	-0.112	0.14
*KLHL3*	Turquoise	-0.618	7.02E-08	0.025	0.33
*SLC2A3*	Blue	0.677	3.02E-05	0.035	0.52

FC: fold-change; FDR: false discovery rate.

### 3.5 Validation of candidate biomarkers by qRT-PCR

To validate the bioinformatics analysis results from the GSE36791 and GSE73378 datasets which indicated that six genes (*BASP1*, *CEBPB*, *ECHDC2*, *GZMK*, *KLHL3*, and *SLC2A3*) are extremely associated with IA rupture, three groups of patients were selected for further analysis of these genes. These groups included patients with IA rupture, patients with a previous aneurysmal SAH (PaSAH), and reference patients. As for reference group, it refers to the patient with no IAs in GSE36791 and GSE73378 datasets. To provide a more reliable validation of these six genes which were differentially expressed between the ruptured and unruptured IA groups [22], we selected the unruptured IA patients to serve as the reference group. When the six biomarkers were assayed by using qRT-PCR, significant differences in expression for all six genes were observed between the ruptured IA group and the reference group (Fig 5C). Specifically, *BASP1*, *CEBPB*, and *SLC2A3* were found to be upregulated, while *ECHDC2*, *GZMK*, and *KLHL3* were downregulated. In contrast, the biomarkers had no validated differences between the PaSAH group and the reference group, except for "*GZMK*" (Fig 5D). These results are consistent with those from the microarray data analysis performed for these selected six genes as described above.

## 4. Discussion

Over the past decade, systems biology approaches have been applied to gene expression datasets with the goal of extracting meaningful information to accompany lists of differentially expressed genes. WGCNA is a widely used data mining method which is especially useful for examining pairwise correlations between variables such as co-expressed genes to provide valuable insights into biological networks [20]. Highly co-expressed genes are connected in a network and can be grouped into modules. In this study, 14 co-expression modules were identified with WGCNA, and then these modules were used to test possible correlations between IAs and clinical traits (i.e., rupture and gender). Within the top three significant modules, the most central and connected genes (the hub genes) were further identified. In a functional analysis of these hub genes, ribosome and related pathways were found to be involved in the rupture of IAs. In our previous study [22], we identified nine key genes associated with IA rupture by screening DEGs of the GSE36791 dataset. In the present study, we established a co-expression network to identify the biological relevance of hub genes by using WGCNA. Six common genes were identified by two methods and were selected from the same dataset. We hypothesize that these six genes which were identified from biologically-relevant hub genes represent valuable biomarkers for the detection and/or treatment of IA rupture.

Among the genes that were found to be upregulated, Brain acid-soluble protein 1 (*BASP1*) was initially identified as an abundant membrane-bound protein in the brain. Accumulating evidence suggests *BASP1* may have roles in neuronal plasticity and axon regeneration [29–31], as well as roles in apoptosis, differentiation, and transcriptional regulation [32, 33]. CCAAT enhancer binding protein beta (*CEBPB*) has been identified as an epigenetic regulator of a mesenchymal signature. *CEBPB* also has important roles in inflammation, cell differentiation, and cell proliferation [34, 35]. In a subset of tumors, abnormal expression and/or methylation of *BASP1* and *CEBPB* have been closely related to occurrence and prognosis. For example, downregulation of *BASP1* expression via promoter methylation may potentially be used to diagnose hepatocellular carcinoma in its early stages [36]. Conversely, *BASP1* is upregulated in cervical cancers and promotes tumorigenicity, thereby identifying it as a novel prognostic factor [29]. In addition, *CEBPB* is highly expressed in glioblastoma stem cells and has been shown to promote glioma progression by regulating cyclin D1 and inducing distinct resistance to chemotherapy [37, 38]. However, there is little known about possible roles and underlying mechanisms for *BASP1* and *CEBPB* in ruptured IAs.

Solute-carrier family 2A3 (*SLC2A3*) encodes the protein, *GLUT-3*, which is a major GLUT subtype for macrophages. GLUT proteins constitute a family of passive glucose transporters which facilitate the uptake of glucose [39, 40]. In abdominal aortic aneurysms (AAAs), Tsuruda et al. [40] demonstrated that *GLUT-3*, as well as *GLUT-1*, appear to be the most important isoforms mediating accumulation of 18 fluoro-deoxyglucose (18F-FDG). In addition, *GLUT-1* and *GLUT-3* immunoreactivity were observed in macrophages in the aneurysmal wall. *GLUT-3* expression has also been associated with metalloproteinase (MMP)-9 activity, which is mainly produced by macrophages in aneurysmal tissues. Correspondingly, high tissue MMP-9 activity has been associated with the progression and rupture of AAAs [41]. Thus, it would be of interest to explore whether there is a similar mechanism for IAs.

Among the genes found to be downregulated, enoyl-coA hydratase domain containing 2 (*ECHDC2*) encodes a mitochondrial protein which contributes to myocardial injury and cell death [42]. Meanwhile, kelch-like3 (*KLHL3*) promotes substrate ubiquitination of bound proteins via interactions of the BTB domain with the cullin 3 component of a cullin-RING E3 ubiquitin ligase complex [43]. Yoshida et al. [44] further demonstrated that mice which present a pseudohypoaldosteronism type II (PHA II) phenotype (i.e., a hereditary hypertensive disease) exhibit a significant decrease in *KLHL3* expression in both kidney and brain tissues, thereby indicating that *KLHL3* may contribute to hereditary hypertension. Since hypertension is an important risk factor for aneurysm rupture, abnormal expression of *KLHL3* may be associated with the onset and rupture of IAs. Granzymes comprise a subgroup of granule-associated serine proteases among a family of immune defense proteases. These granzymes have important roles in both innate and adaptive immunity [45, 46]. In humans, there are five granzyme genes (*GZMA*, *GZMB*, *GZMH*, *GZMK*, and *GZMM*). Similar to granzyme A, granzyme K (*GZMK*) is a pro-apoptotic serine protease which is secreted from granules. It has been demonstrated that *GZMK* exhibits pro-inflammatory potential by inducing the secretion of multiple inflammatory factors such as interleukin (IL)-1β, IL-6, IL-8, and monocyte chemoattractant protein-1 (MCP-1). In addition, *GZMK* activates protease-activated receptor-1 to induce cell proliferation [46–48].

Gene expression profiles of the six genes of interest were validated by using the GSE73378 dataset. There were no significant differences in gene expression levels between the PaSHA and the reference groups, and this was confirmed with qRT-PCR. Overall, the results obtained indicate that *BASP1*, *CEBPB*, and *SLC2A3* are selectively upregulated, while *ECHDC2*, *GZMK*, and *KLHL3* are downregulated, in patients with ruptured IAs compared to the reference group. Furthermore, all of the genes, except *GZMK*, exhibited the same expression pattern in the PaSHA and reference samples. Based on these results, we have demonstrated that these six genes undergo dynamic changes in IAs during the transition from an unruptured stage to a recovery stage (Fig 5E). Correspondingly, we detected increases or decreases in gene expression among these six gene targets during the IA rupture stage, and it is predicted that these levels return to “normal” during the subsequent recovery stage. Therefore, the potential for these six genes to serve as biomarkers for predicting IA rupture may have important clinical implications. Moreover, whether there is a threshold interval of gene expression which can distinguish unruptured IAs from ruptured IAs is also important to consider. Thus, monitoring of these biomarkers in peripheral blood samples from patients with unruptured IAs could provide a guide for clinical treatment.

There were several potential limitations associated with the present study. First, our datasets were downloaded from the GEO database, and thus, some clinical information is absent (e.g., patient age, cigarette smoking status, hypertension, and size and location of IAs). Second, a relatively small sample size was validated by qRT-PCR. Based on these two potential limitations, a prospective observational cohort study will be conducted to demonstrate the potential prediction function of these six biomarkers, with qRT-PCR (mRNA) and detection of proteins in plasma used to characterize the biomarkers. Briefly, we will serially and longitudinally track patients who progress from an unruptured IA to a ruptured IA, and then post-rupture. Ideally, appropriate genetic data, as well as clinical status data, will be collected for a large number of patients.

In conclusion, we used bioinformatics to analyze datasets generated from patients with ruptured IAs in order to identify hub genes which are associated with the progression and rupture of IAs. A total of 238 hub genes exhibited high connectivity with IA rupture. In particular, *BASP1*, *CEBPB*, *SLC2A3*, *ECHDC2*, *GZMK*, and *KLHL3* were identified and validated by qRT-PCR. Based on these results, we hypothesize that these six genes potentially represent valuable biomarkers for predicting IA rupture, and their validation would improve both clinical management and treatment outcome of patients with unruptured IAs.

## Supporting information

S1 Fig Directed acyclic graph of biological process (BP) terms. (TIF)

S2 Fig Directed acyclic graph of cellular compartment (CC) terms. (TIF)
