# Supplementary figures and images for: Weighted gene co-expression network analysis identified six hub genes associated with rupture of intracranial aneurysms
Source: PLoS One. 2020 Feb 21;15(2):e0229308. doi: 10.1371/journal.pone.0229308 (PMC7034829; doi:10.1371/journal.pone.0229308)

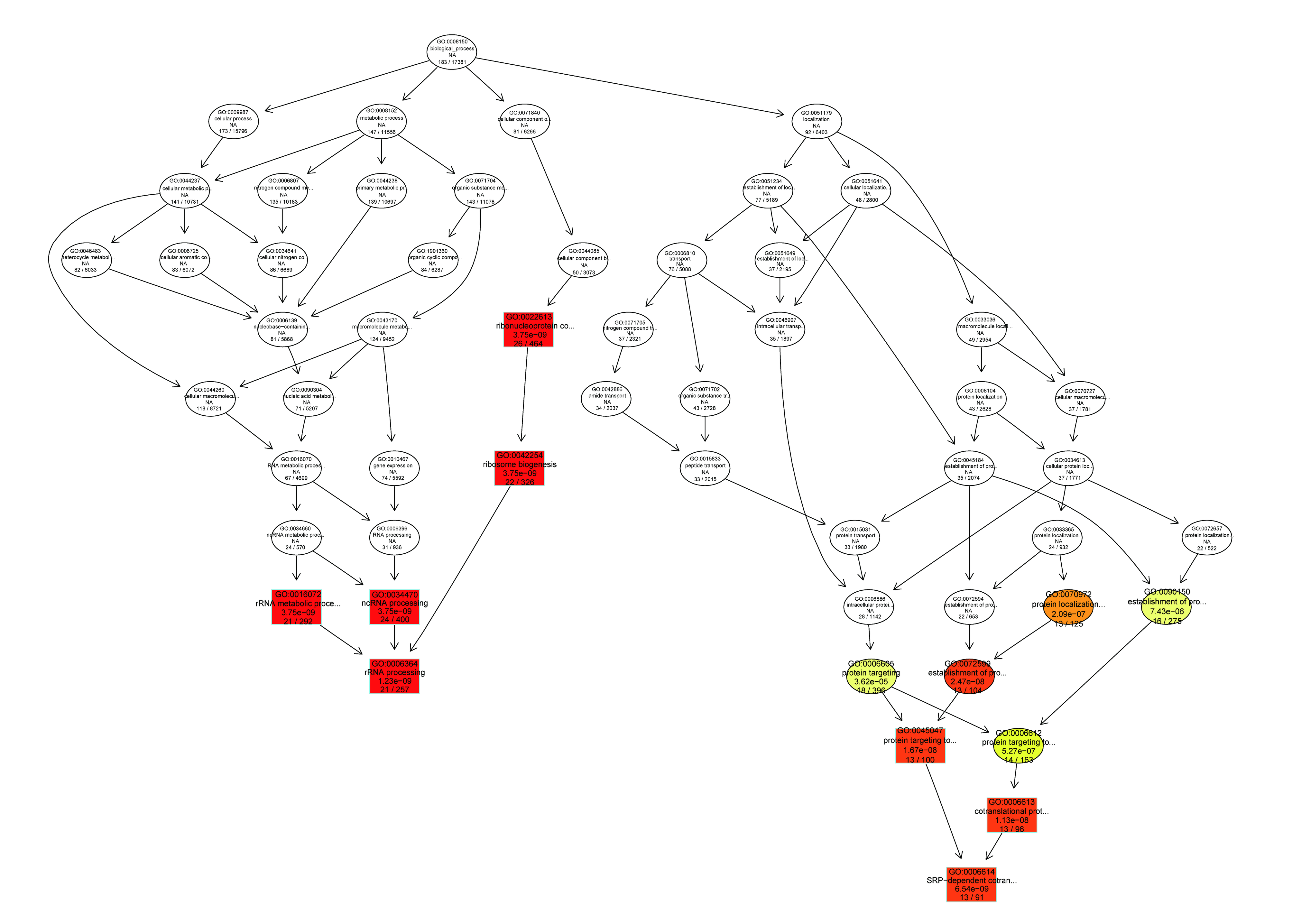

Supplement: S1 Fig — (TIF) [file pone.0229308.s001.tif]

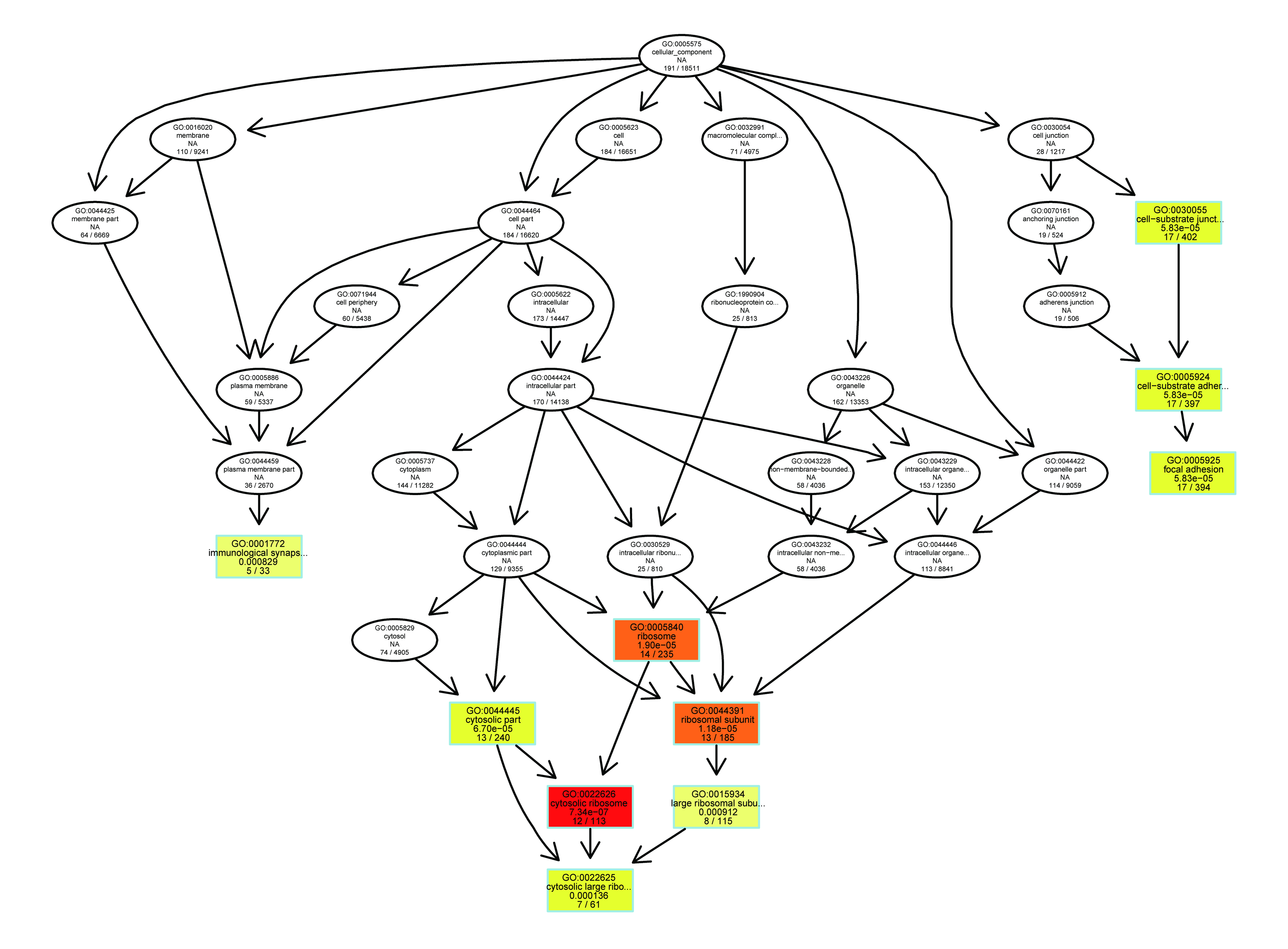

Supplement: S2 Fig — (TIF) [file pone.0229308.s002.tif]
